# Melatonin-Medicated Neural JNK3 Up-Regulation Promotes Ameloblastic Mineralization

**DOI:** 10.3389/fcell.2021.749642

**Published:** 2021-12-24

**Authors:** Qianhui Ren, Jing Pan, Yunshuo Chen, Zhecheng Shen, Zhao Yang, Kubin Kwon, Ying Guo, Yueying Wang, Fang Ji

**Affiliations:** ^1^ Shanghai Key Laboratory of Stomatology, National Center for Stomatology, National Clinical Research Center for Oral Diseases, Department of Orthodontics, Shanghai Ninth People’s Hospital, Shanghai Jiao Tong University School of Medicine, College of Stomatology, Shanghai Jiao Tong University, Shanghai, China; ^2^ State Key Laboratory of Medical Genomics, National Research Center for Translational Medicine at Shanghai, Rui Jin Hospital, Shanghai Institute of Hematology, Shanghai Jiao Tong University School of Medicine, Shanghai, China

**Keywords:** amelogenesis imperfect, mineralized tissue/development, signal transduction, Arrb1-JNK3 signaling, RNA sequencing

## Abstract

**Introduction: **Melatonin, an endogenous neurohormone, modulates the biological circadian rhythms of vertebrates. It functions have been reported in previous stomatological studies as anti-inflammation, antioxidant, osseointegration of dental implants and stimulation to dental pulp stem cells differentiation, but its role in ameloblastic differentiation and mineralization has been rarely studied.

**Objective: **To reveal the effects of melatonin on the mineralization of ameloblast lineage cells (ALCs), and to identify the change in gene expression and the potential mechanism based on ribonucleic acid sequencing (RNA-seq) analysis.

**Method: **ALCs were induced in melatonin-conditioned medium. After 7-days culture, Western blot, real-time PCR, alkaline phosphatase (ALP) activity test, RNA-seq were accordingly used to detect the change in molecular level. After 1-month odontogenic induction in melatonin medium, Alizarin Red-S (ARS) staining showed the changes of mineral nodules. Differentially expressed genes (DEGs), enrichment of functions and signaling pathways analysis based on Kyoto Encyclopedia of Genes and Genomes (KEGG) and Gene Ontology (GO) database were performed. The JNK3 antagonist (JNK3 inhibitor IX, SR3576) and β-arrestin1 (Arrb1) overexpression were applied to confirm the fluctuation of melatonin-medicated JNK3 and Arrb1 expression.

**Results: **In this study, we found out melatonin contributed to the ameloblastic mineralization, from which we can observed the elevated expression of enamel matrix protein, and increased ALP activity and mineralized nodules formation. RNA-seq analysis showed the up-regulation of neural JNK3 and down-regulation of Arrb1 in ALCs. Meanwhile, phosphorylated JNK3 deficiency (phosphorylated JNK3 inhibitor---SR3576 added to culture medium) led to mineralization delay, and Arrb1 overexpression proved Arrb1 takes bridge between melatonin receptors (MTNR) and JNK3 in MAPK signaling pathway.

## Introduction

Amelogenesis imperfecta (AI) is an inherited developmental enamel defects, with enamel abnormally thin, fragile and/or discolored (Al-Abdallah et al., 2015). AI enamel usually has poor function and aesthetics, causing patients problems such as premature tooth loss, severe social embarrassment, eating difficulties, and pain (Negre-Barber et al., 2018). Mature enamel is the hardest, most mineralized tissue in the human body, comprising >95% by weight crystals of substituted calcium hydroxyapatite (HA; Ca_10_ [PO_4_]_6_ [OH]_2_), which is secreted by the ameloblasts, the enamel-forming cells (Lacruz et al., 2017). The ameloblasts are lost upon tooth eruption. Consequently enamel lacks any capacity for cellular repair and once formed, must function over a lifetime (Smith et al., 2017). In view of the non-renewable nature of enamel, it is urgent and necessary for us to study the mechanism of amelogenesis imperfect.

In dental enamel, there are two regularly occurring incremental markers: daily cross-striations and long-period striae of Retzius (SR lines), suggesting the evidence for periodic growth of enamel (Kierdorf et al., 2019). Given the existence of enamel periodicity, we focused on the two stimuli that significantly regulate the biological rhythm of mammals, photoperiod and melatonin. Our previous studies revealed for the first time that melatonin-mediated circadian rhythm disturbance caused extensive degeneration of ameloblasts, low mineralization of enamel and significantly reduced amelogenin (AMELX) content in the first molar tooth embryo of 7 and 14 days postnatal mice (Tao et al., 2016). Circadian rhythms in mammals are regulated globally by the master clock in the suprachiasmatic nucleus (SCN), and locally by clock cells that control tissue-specific rhythmic outputs (Simmer et al., 2010). This central rhythm generator, in turn, sends a stimulus to the pineal gland, activating the endocrine melatonin synthesis pathway. Melatonin and its downstream pathways transmit central signals to the periphery (Adeola et al., 2019). Thus, there is sufficient evidence that there might be a strong link between circadian rhythm and melatonin. By now, melatonin has also been proven to be relevant to regulating body temperature (Permuy et al., 2017), acting as an anti-aging molecule (Hardeland et al., 2011) and a neuroendocrine regulator for multiple organs (Slominski et al., 2012). As to stomatological functions, melatonin contributes to osteointegration of dental implants (Calvo-Guirado et al., 2010), anti-inflammation in dental pulp (Kantrong et al., 2020), and somehow alters the proliferation of cell lines in a dose-dependent manner and prevents early dentin formation *in vivo*, while SCN plays an important role in generating circadian dentine increments *in vivo* (Liu et al., 2013).

Researchers have found many probable causes (X-linked AMELX gene mutation (Smith et al., 2017), disordered interaction between odontogenic epithelium and mesenchyme (Jiang et al., 2017), function of ion transportation (Josephsen et al., 2010), et al.), trying to explain the underlying mechanism of AI, whereas melatonin-medicated circadian rhythm was rarely involved. And according to our previous study, there are a good deal of mechanism waiting to be explored. Thus, this study delves into the underlying mechanism of melatonin-medicated enamel defect.

## Materials and Methods

### Reagents and Cell Lines

Professor Nakata and his co-worker isolated ALCs from tooth germs of newborn C57BL/6J mouse mandible molars, which is a spontaneously immortalized cell line, maintaining the cell type specific character—the ability to induce *in vitro* bio-mineralization (Sarkar et al., 2014). ALCs is a proper tool for enamel mineralization research (Nakata et al., 2003). Melatonin, JNK3 inhibitor SR3576, MTNR1a&MTNR1b inhibitor Luzindole (LUZ), Alizarin Red-S was purchased from Sigma. Antibodies against Arrb1, β-Actin, proliferating cell nuclear antigen (PCNA) and HRP-linked anti-rabbit IgG were from Cell Signaling Technology. Anti-total JNK3, AMELX, ameloblastin (AMBN), odontogenic ameloblast-associated protein (ODAM) was from Abcam. Phos binding reagent acrylamide was purchased from APE×Bio.

### Cell Culture

ALCs were seeded and passaged in 4.5 g/L D-glucose Dulbecco’s Modified Eagle Medium (DMEM; Gibco) supplemented with 10% fetal bovine serum (FBS; Gibco), with 10,000U/ml penicillin and 10,000 μg/ml streptomycin (Gibco, Australia). Cultured cells were incubated at 37°C in a humid atmosphere of 95% air and 5% CO_2_ for 7 days. Melatonin was supplemented in DMEM with concentration of 1 mM, 0.1 mM, 0.1 μM, and 1 nM, LUZ with concentration of 10 μM, 1 μM, 1 nM, and 10 pM accordingly, SR3576 with concentration of 25 and 50 μM.

### Odontogenic Induction and Alizarin Red-S Staining

Cells were cultured in 6 cm-diameter dishes in 5% CO_2_ at 37°C. After 24-h attachment, the normal DMEM was changed to the odontogenic induction medium, containing 1 g/L D-glucose DMEM with 10% FBS, 5 mM β-glycerophosphate (Sigma, United States), 50 μg/mL L-ascorbic acid (Sigma, United States) and melatonin/LUZ/SR3576 of different concentration. Cultures were maintained for an additional 1 month with a medium change every 3 days. Then, cells were rinsed in PBS, fixed in a solution of 4% paraformaldehyde, and stained with 40 nM Alizarin Red-S solution (PH = 4.2). The dishes were washed with ddH_2_O four times to remove the nonspecific staining. The photos of mineralized nodules were taken in digital camera and inverted phase contrast microscope (IPCM). The calcified nodules were extracted by 10% Cetylpyridinium Chloride and measured at 562 wavelength.

### Quantitative Real Time PCR Analysis

Total mRNA was extracted by EZ-press RNA Purification Kit (EZbio science, United States), and concentrations were measured by Nanodrop-800, followed by reverse transcription to synthesize cDNA. First-strand cDNA was synthesized using the PrimeScript™ RT Master Mix (TaKaRa, Japan). qPCR system was prepared using SYBR Green I Mastermix (Roche Applied Science, Germany). The qPCR reaction conditions were set according to operating instruction: 1 cycle at 95°C for 5 min, followed by 40 cycles at 95°C for 10 s, 60°C for 20 s, and 72°C for 20 s. Ct values were processed using the 2-ΔΔCT method. The relative expression level of each gene was normalized to β-actin. The sequences of primers (forward/reverse) were listed in Supplementary Tables S1, S2.

### Western Blotting

Cells were homogenized in RIPA buffer (ThermoFisher, United States) containing complete protease inhibitor and phosphatase inhibitors (Roche), and a BCA assay kit (Beyotime, China) was used to determine total protein concentration. Then, samples were further diluted in 5× Loading buffer (Beyotime, China) and boiled for 30 min. Equal amounts of protein (20 μg of total protein) from each sample were loaded and ran on 8–16% Expressplus PAGE gels (Genscript) and transferred to PDVF membranes (Merck, United States). After blocking in QuickBlock Blocking Buffer (Beyotime, China) at room temperature, the membranes were probed with primary antibodies overnight at 4°C respectively. A control blot incubated with β-actin antibody was used to normalize the amount of protein. The membrane was washed and then probed with HRP-linked anti-rabbit secondary antibody for 1 h at RT, followed by ECL detection (Merck, United States). The intensities of the bands were scanned and measured with Fiji (Schindelin et al., 2012). Specifically, phos binding reagent acrylamide was added to PAGE gels in concentration of 50 μM.

### Alkaline Phosphatase Activity Test

Alkaline phosphatase Assay Kit (Beyotime Biotechnology, China) was used to perform ALP activity at day 7 according to the manufacturer’s instruction by the PNPP method. Cultured cells were lysed by lysis buffer (Beyotime Biotechnology, China) for 5 min, and then were incubated with fresh *p*-nitrophenol at 37°C for 1 h. The optical density was read spectrophotometrically at 405 nm wavelength. Relative ALP activity was normalized by the total protein concentration.

### RNA Sequencing and Bioinformatics

Total RNA was extracted from ALCs stimulated with or without 0.1 mM melatonin. RNA purified using Ribo-Zero rRNA Removal Kit. The concentration and purity of total RNA were measured with ND-800 spectrophotometer. RNAs libraries were constructed with Truseq™ RNA Library prep Kit. Sequencing was carried out using a 2 × 150 bp PE configuration. The clean data (reads) were mapped using Hisat2 (version 2.1.0). Then, we performed gene expression analysis using RNA-seq by Expectation Maximization (RSEM, version 1.3.1). Differential expression analysis among different groups were analyzed using DESeq2, and then fold change >2 and false discovery rate (FDR) < 0.05 were determined as thresholds to different expression genes. DEGs were validated by RT-PCR. GO analysis was used to calculate the most significant functions of a particular gene set (q < 0.05). Venn analysis was used to calculate the number of genes/transcripts in each gene set and the overlap relationship between different gene sets. Biological Pathway enrichment analysis was based on KEGG pathway database. KEGG terms with q < 0.05 were considered to be significantly enriched. To validate the reliability of the DEG results, the expression levels of 12 selected transcripts were determined by qRT-PCR with β-Actin as an endogenous reference. Another 12 candidate genes primers were listed in (Supplementary Tables S2).

### Accession Number

The database presented in this study can be found in online repositories. The names of the repository and accession number can be found below: Sequence Read Archive (SRA) of NCBI, accession number: PRJNA770009.

### Recombinant Lentivirus Vector Construction and Package

Full-length Arrb1 (NM_177,231.2, 1257 bp) was cloned into expression vector GM-18458 (Genomeditech, China) bearing mScarlet reporter and the second selection gene encoding an puromycin N-acetyl-transferase gene, which conferred resistance to puromycin. The letivirus was produced by a cotransfection with the psPAX2 packaging plasmid and the pMD2. G envelop plasmid. Recombinant lentivirus was harvested 72 h following cotrasfection of three vectors into 293T cell at the indicated concentrations using Lipofectamine 2000 (Invitrogen Life Technologies) according to the manufacturer’s instructions. The virus supernatant was purified, and the viral titer was determined.

### Lentiviral Vector Transduction and mScarlet Reporter Gene Detection

The ALCs (1 × 10^6^ per well) were seeded in six-well culture plates and cultured for 24 h. The lentivirus was then added at a MOI value of 100:1. After 24 h of coculture, the stable cell lines were selected with 3 mmol/L puromycin (Merck, United States). The puro-resistant ALCs were then collected and cultured in normal culture media for 20 passages. The percentage of red fluorescence positive cells were evaluated by fluorescence microscopy.

### Immunofluorescence

ALCs and overexpression cell lines were plated on glass bottom cell culture dishes (Nest, China) (overexpression cell lines’ medium containing 1.5 mg/L maintain puro). After 7 days, cells were washed with PBS, fixed with 4% paraformaldehyde solution, and permeabilized with 0.1% Triton X-100. Cells were then incubated with the anti-JNK3 and secondary antibody (Abcam, United States). Images were acquired on a Zeiss (Jena, Germany) LSM 510 Meta confocal microscope.

### Statistical Analysis

Statistical tests were performed by GraphPad Prism (version 7.02). The data were presented as mean ± SD. Student’s t-test was used for the comparison between the two groups. One-way analysis of variance (ANOVA) followed by Bonferroni’s test was used for multiple group comparisons. A value of *p* < 0.05 was considered statistically significant. All experiments were independently repeated in 3–6 times.

## Result

### Melatonin Promotes Ameloblastic Mineralization

**FIGURE 1:**
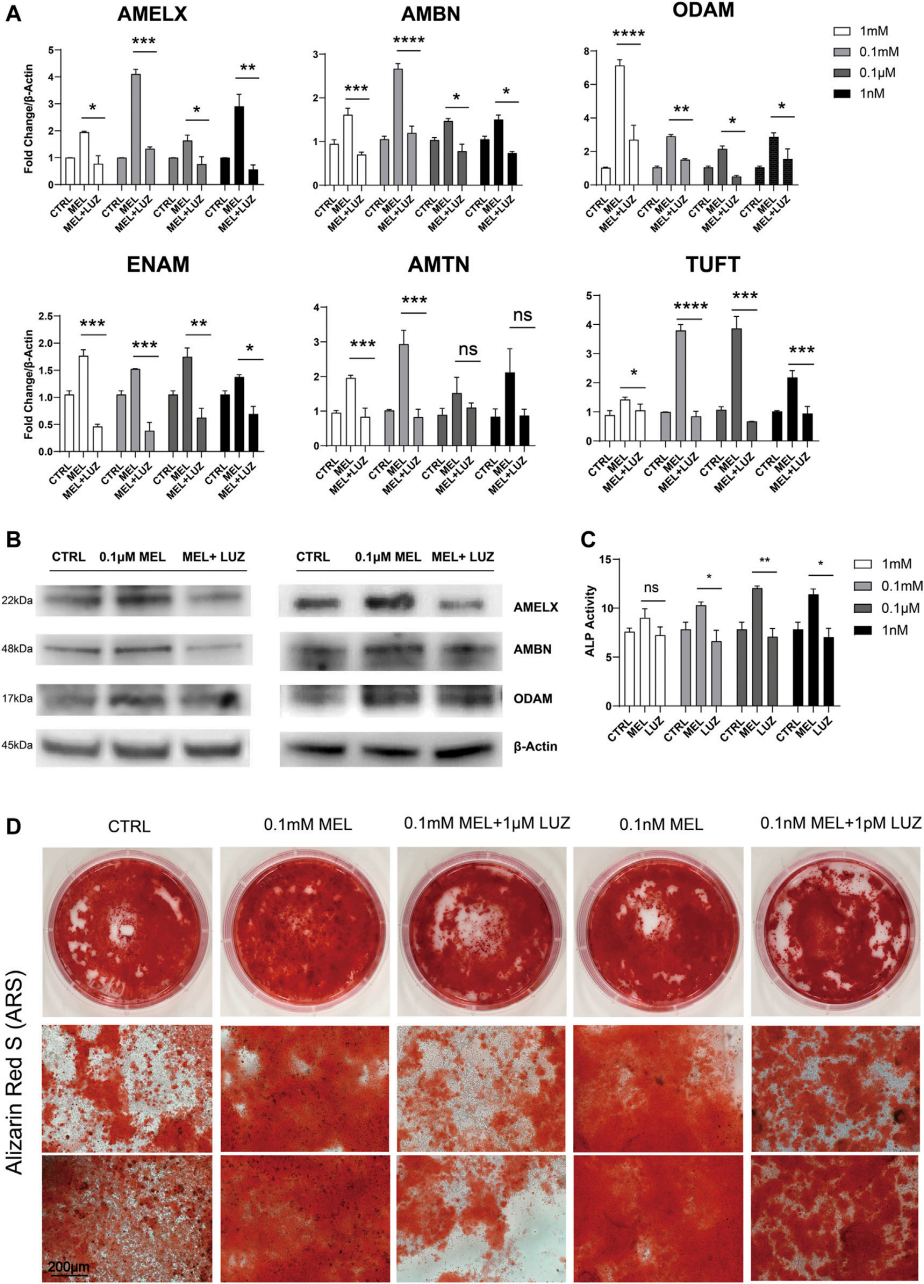
Melatonin promotes ameloblastic mineralization. **(A)** The relative mRNA level of EMPs (AMELX, AMBN, ODAM, ENAM, AMTN, and TUFT)*. ALCs were cultured in different concentration of melatonin/melatonin + luzindole. The horizontal lines at the top of MEL and MEL + LUZ group mean that the pairwise comparison between (1) CTRL group and MEL group, and (2) MEL and MEL + LUZ group (mean ± SD; five independent experiments). **(B)** Protein level of EMPs (AMELX, AMBN, and ODAM). ALCs were from melatonin- and/or luzindole-supplemented medium (0.1 mM, 0.1 μM). Protein concentration was normalized by β-Actin. **(C)** ALP activity of Cells cultured in 1 mM, 0.1 mM, 0.1 μM, and 1 nM melatonin medium with **(right columns)** or without **(middle columns)** corresponding luzindole. The ALP concentration was normalized by *p*-nitrophenol (mean ± SD; five independent experiments). **(D)** Alizarin Red-S staining after 1-month melatonin/luzindole induction. ALCs were from melatonin- and luzindole-supplemented odontogenic medium (0.1 mM, 0.1 μM). The magnification of IPCM was 200 μm. *p < 0.05 (one-way ANOVA and LSD test). *Abbreviation information: AMELX-amelogenin, AMBN-ameloblastin, ODAM-odontogenic ameloblast-associated protein, ENAM-enamelin, TUFT-tuftelin, and AMTN-amelotin.

We found melatonin-medicated ALCs robustly produced enamel matrix proteins (EMPs) in mRNA level (Figure 1A) especially in 0.1 mM group, suggesting that melatonin stimulates the ALCs to produce amelobalstic scaffolding proteins, and 0.1 mM maybe the optimal concentration. At the meantime, after blocking G-protein-coupled MTNR by luzindole, it showed that luzindole obviously reduced the mRNA level of EMPs, comparing to melatonin group. Thus, we selected the optimal 0.1 mM and less optimal 0.1 μM to verify the changes in protein (Figure 1B), showing that melatonin-induced group significantly up-regulated protein expression, but luzindole reversed this change. Meanwhile, melatonin induced ALCs to elevate the ALP activity (Figure 1C). After 30 days induction in odontoblastic medium, melatonin accelerated the process of mineralization, and comparing to control group, the melatonin group presented a much more numerous and more dyeing mineralized nodule (Figure 1D and Supplementary Figure S1A). Similarly, luzindole reduced the degree of mineralization, which confirmed melatonin stimulate amloblastic differentiation and mineralization through melatonin receptor.

### RNA Sequencing Analysis

**FIGURE 2:**
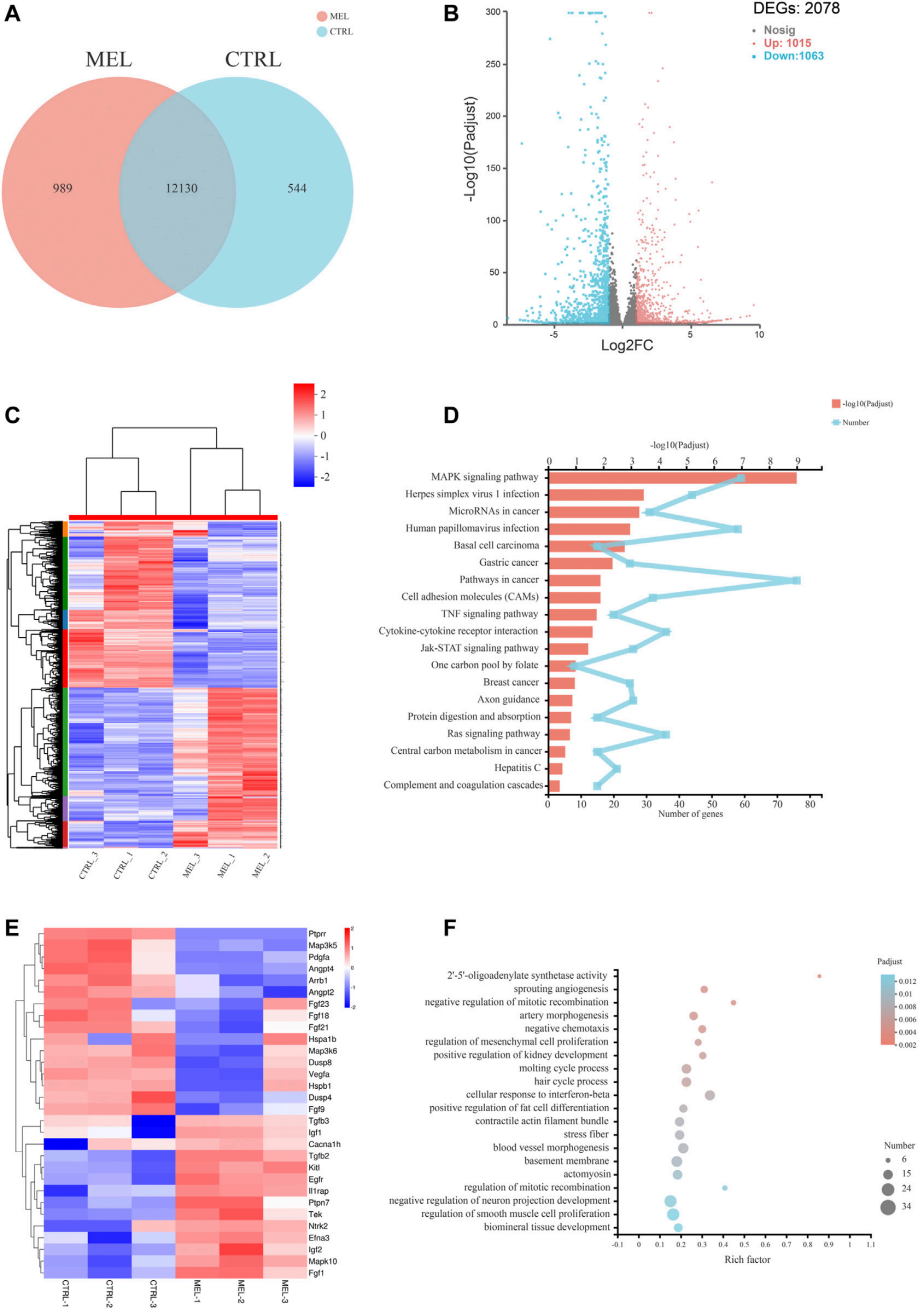
RNA sequencing Analysis. **(A)** Venn diagram: unique and overlapping gene expression between CTRL and MEL. **(B)** Volcano map of DEGs between ALCs medicated with and without melatonin (MEL group and CTRL group). The *x*-axis is the log2FC of gene expression. Negative values (blue): down-regulation; positive values (red): up-regulation. The *y*-axis is–log10 (Padjust), which indicate the significant level of expression difference. **(C)** Hierarchical clustering heat map of mRNA signatures in two groups. Each column represents a sample (CTRL1-3, MEL1-3). The color in the graph indicates that the gene expression pattern. Red, up-regulated; blue, down-regulated. On the left is the tree diagram of gene clustering and the module diagram of subclustering. On the right is the name of the gene. **(D)** The top 20 enriched KEGG pathway terms of DEGs. Padjust<0.05. **(E)** Heat map of DEGs (log2FC > |2|) in MAPK signaling pathway. **(F)** Significant enriched GO terms between CTRL group and Mel group. The vertical axis represents GO Term, and the horizontal axis represents the ratio of the Rich Factor. The size of the dot denotes the number of genes in the GO Term, and the color of the dot corresponds to different Padjust ranges.

Basic Analysis To reveal the molecular mechanism associated with melatonin-medicated ameloblastic differentiation, we sequenced the transcriptome of control groups and melatonin-medicated groups (3 samples in each group). The Venn diagram (Figure 2A) showed a unique and overlapping gene expression pattern between two groups, with 12130 overlapping expressed genes and 989 unique-expressed genes in MEL group, 544 in CTRL group respectively. The Hierarchical clustering heat map (Figure 2B) of three replicates of CTRL and MEL samples exhibited highly consistent transcriptional changes. Thus, a DEG analysis was performed to identify gene expression changes between control group and 1 mM melatonin-treated group. A total of 2078 DEGs (log_2_FC > 2 and p-adjust < 0.05) were detected, of which 1,015 genes were up-regulated (higher expression in treatment group) and 1,063 genes were down-regulated (Figure 2C).

Functional annotation and classification KEGG pathway enrichment analysis of all DEGs was conducted to explore a statistically significant enrichment for 20 pathways for DEGs (Figure 2D). The most enriched pathway was MAPK signaling pathway. All DEGs (log2FC>|2|) associated with the MAPK pathway were listed in the heat map (Figure 2E), and the Ntrk2, Mapk10, Efna3, Fgf1, Ptpn7, Fgf21, Fgf23, Angpt4, and Ptprr were significantly up- or down-regulated (log2FC > |2.5|), indicating the activation of the melatonin-medicated MAPK signaling pathway during ameloblastic differentiation. The top three GO enrichment pathways are 2′-5′-oligoadenylate synthetase activity, sprouting angiogenesis, negative regulation of mitotic recombination (Figure 2F).

### Validation of the Sequencing Results by qRT-PCR

**FIGURE 3:**
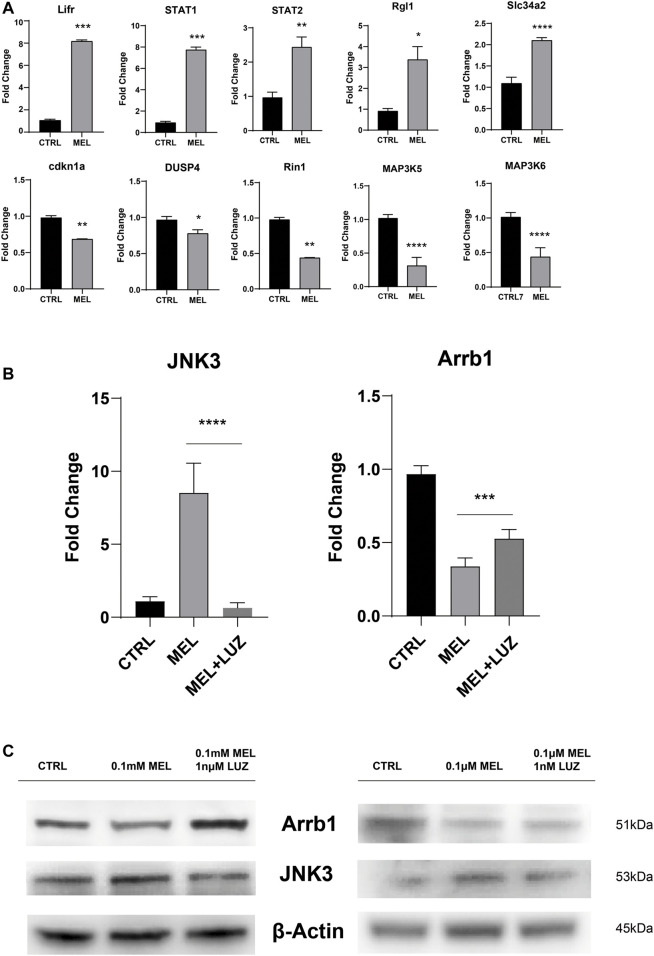
Validation of the sequencing results by qRT-PCR **(A)** The relative mRNA level of DEGs. Total mRNA was obtained from RNA-seq samples (mean ± SD; five independent experiments). **(B)** The relative mRNA level of JNK3 and Arrb1 (mean ± SD; five independent experiments). **(C)** Total proteins probed by anti-JNK3 and anti-Arrb1 antibodies. Protein concentration was normalized by β-Actin. All the experiments have been repeated 3–6 times. *p < 0.05 (one-way ANOVA and LSD test).

To confirm the reliability of the expression profiles, qRT-PCR (Figure 3A,B) was applied to examine the expression levels of the 12 candidate genes (up-regulated: Lifr, STAT1, STAT2, Rgl1, Slc34a2, and JNK3; down-regulated: cdkn1a, DUSP4, Rin1, MAP3K5, MAP3K6, and Arrb1). As expected, the qRT-PCR results basically matched the RNA-seq results. The results indicate that the RNA-seq data reliably identified potential genes in ALC ameloblastic differentiation and mineralization.

JNK3 (MAPK10, a mitogen-activated protein kinase) showed a trend of up-regulation both in mRNA and protein levels (Figure 3B,C). According to GO term, JNK3 participate in biological process of “signal transduction (GO id:GO:0007165)”, “regulation of gene expression (GO id:GO:0010468)”, “regulation of circadian rhythm (GO id:GO:0042752)”, et al. And Arrb1 was involved in process of “activation of MAPK activity (GO:0000187)”, “G-protein coupled receptor internalization (GO:0002031)”, “signal transduction (GO:0007165)”.

### Jun N-terminal Kinase3 Phosphorylation Inhibitor (SR3576) Repressed the Melatonin-Medicated Differentiation and Mineralization

**FIGURE 4:**
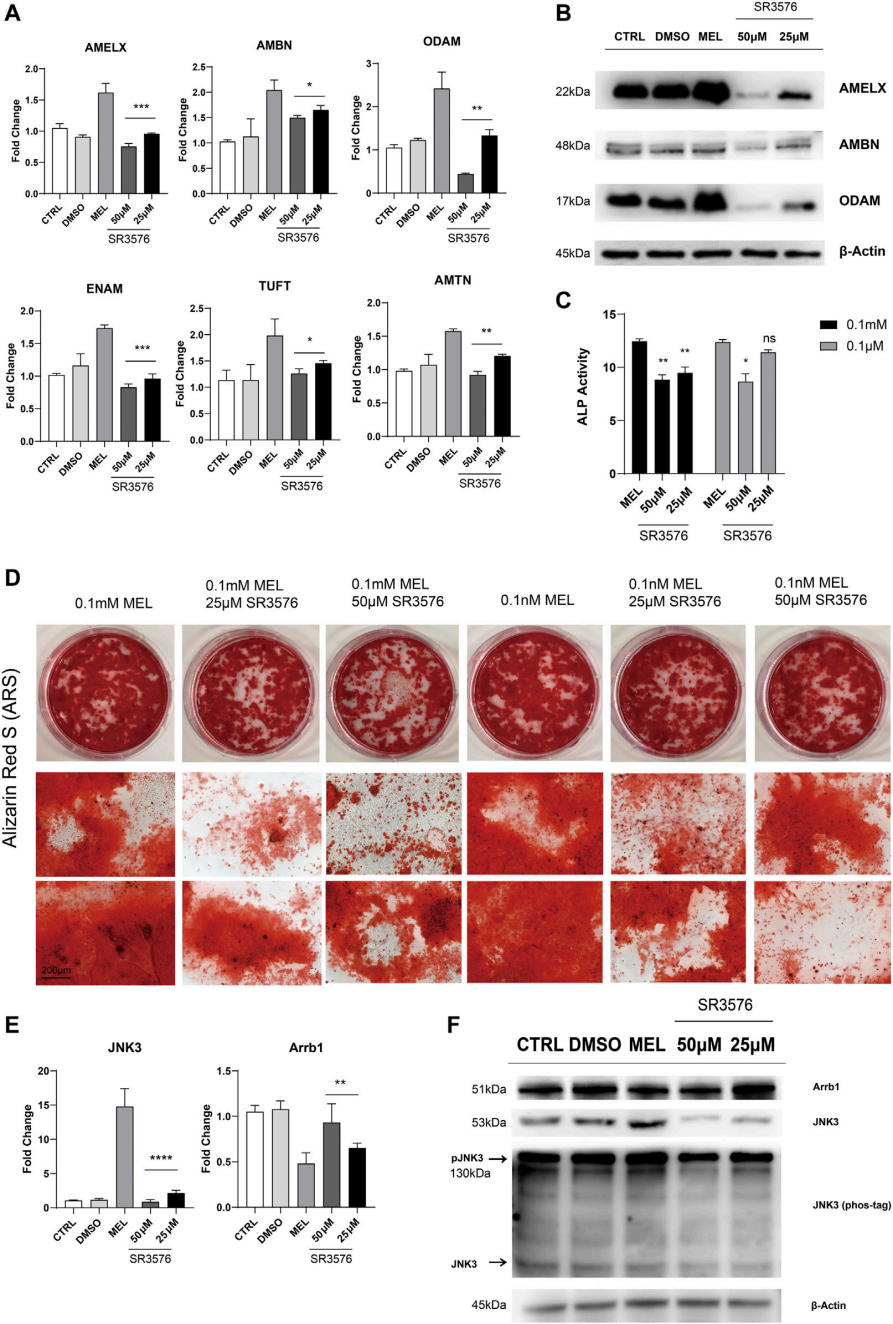
JNK3 phosphorylation inhibitor SR3576 repressed the melatonin-medicated differentiation and mineralization. **(A)** The relative mRNA level of EMPs (AMELX, AMBN, ODAM, ENAM, AMTN, and TUFT). ALCs cultured in different concentration of SR3576 (0.1 mM melatonin based medium) was used for qRT-PCR. The horizontal lines at the top of 25 and 50 μM SR3576 group mean that the pairwise comparison between (1) 0.1 mM MEL and 25 μM group, and (2) 0.1 mM MEL and 50 μM group (mean ± SD; five independent experiments). **(B)** Total proteins extracted from melatonin- and SR3576-medicated ALCs probed by anti-AMELX, anti-AMBN and anti-ODAM anti-JNK3 antibodies. Protein concentration was normalized by β-Actin. All the experiments have been repeated 3–6 times. **(C)** ALP activity from cells in 0.1 mM melatonin medium with **(right columns)** or without **(middle columns)** 25μM/50 μM SR3576 (mean ± SD; five independent experiments). **(D)** Alizarin Red S staining after 1-month melatonin with or without 25μM/50 μM SR3576 odontogenic induction. The magnification of IPCM was 200 μm. **(E)** The relative mRNA level of JNK3 and Arrb1. ALCs were cultured in different concentration of SR3576 (0.1 mM melatonin based medium). **(F)** Proteins levels from melatonin- and SR3576-medicated ALCs probed by anti-JNK3, anti-Arrb1 antibodies and phos-tag. *p < 0.05 (one-way ANOVA and LSD test).

In order to confirm melatonin-medicated function, we added JNK3 phosphorylation inhibitor (SR3576) to melatonin-based medium, resulting in decreasing enamel matrix protein both in mRNA and protein level (Figure 4A,B), lower ALP activity (Figure 4C) and reductive and less dyed mineralization nodules (Figure 4D and Supplementary Figure S1B), indicating JNK3 contributed to mineralization process. According to RNA sequencing, qRT-PCR, Western blotting results, Arrb1 showed a significant down-regulation in MEL group, and we arouse a hypothesis that Arrb1 may take bridge in MTNR-MAPK signaling pathway, whose down-regulation may accelerate the process of differentiation and mineralization.

### Arrb1 Overexpression Contributed to Jun N-terminal Kinase3 Nuclear Accumulation

**FIGURE 5:**
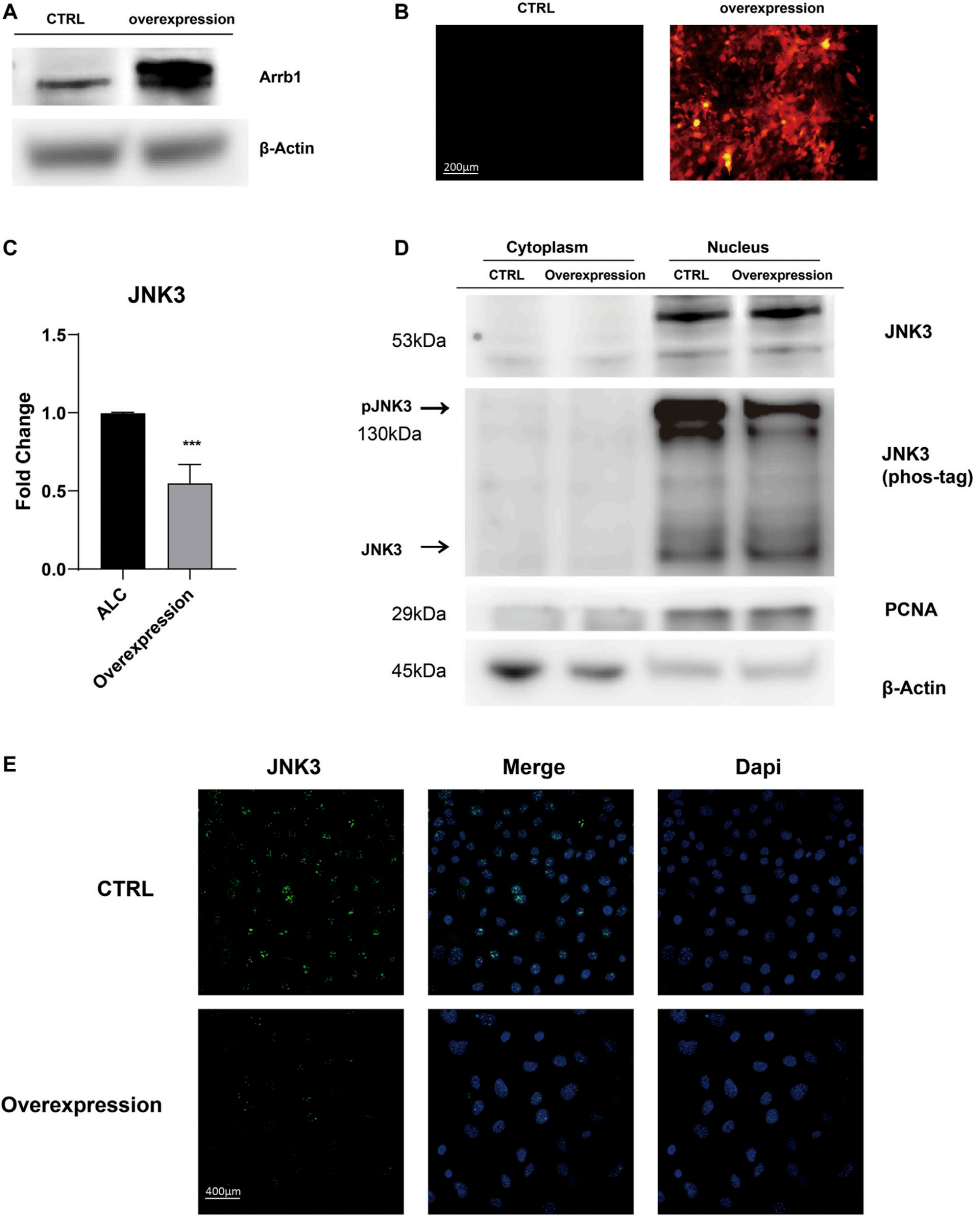
Arrb1 overexpression contributed to JNK3 nuclear accumulation **(A)** Proteins levels from non-transfection and overexpressed ALCs probed by anti-Arrb1 antibodies. Protein concentration was normalized by β-Actin. **(B)** Non-transfected and stably-overexpressed ALCs observed under a fluorescence microscope (200 μm magnification). **(C)** The relative mRNA level of JNK3. Non-transfected and stably-overexpressed ALCs was used for qRT-PCR (mean ± SD; five independent experiments). *p < 0.05 (one-way ANOVA and LSD test). **(D)** Cytoplasmic and nuclear proteins extracted from non-transfection and overexpressed ALCs probed by anti-JNK3 antibodies and phos-tag. Protein concentration was normalized by β-Actin and PCNA. All the experiments have been repeated 3–6 times. **(E)** Immunofluorescence of JNK3 in non-transfected and Arrb1 stably-overexpressed ALCs. The magnification is 400 μm.

After Arrb1 lentiviral vector was transferred (Figure 5A,B), stably overexpressed ALCs were selected by puromycin. We found Arrb1 overexpression down-regulated the mRNA levels of JNK3 (Figure 5C), and at the same time, we isolated the nuclear protein from cytoplasmic, WB results showing that JNK3 mainly distributed in nucleus. The up-regulated expression of Arrb1 conversely inhibited the phosphorylation of JNK3 (Figure 5D). We can observed the nuclear location of JNK3 (Figure 5E).

## Discussion

Amelogenesis Imperfecta is a common and frequently-occurring disease in oral clinic. With the emergence and enrichment of the by-products of economic growth and social development (Reed et al., 2020), the incidence of tooth defect is rising year by year. Therefore, it is of great clinical significance to further study the pathogenesis of enamel hypoplasia and to find out effective preventive measures.

In the area of molecular developing biology, melatonin is referred to tooth development, by regulating cellular processes in odontogenic cells (Permuy et al., 2017), altering mitochondrial activity in dental papilla cells (Liu et al., 2013; Jiang et al., 2019), et al. In previous study, we have discovered the gene and protein expression of MTNRs in ALCs (Supplementary Figure S1C,D). What’s more, mounting evidence in this study indicated that melatonin does promote the mineralization of ALCs through melatonin receptor dependent pathway *in vitro*, for instance, stimulating the expression of EMPs and the activity of ALP, promoting the deposition of calcified nodules. In mammals, MTNR-dependent pathway contains MTNR1a and MTNR1b. Luzindole is well accepted in blocking both MTNR1a and MTNR1b in the meantime (Gengatharan et al., 2021). Therefore, we used Luzindole to study the melatonin effect via MTNR-dependent pathway. EMPs are distributed in the extracellular matrix and play an important role in mineralization and differentiation. Once synthesis is reduced or impeded, the enamel may be underdeveloped (Crawford et al., 2007). ALP is an important enzyme in whole process of mineralization (Li et al., 2019a), commonly used in odontogenic and osteogenic differentiation and calcification (Ching et al., 2017). In our experiment, ALP and ARS were used as short-term and long-term indicators for mineralization. The research between melatonin and AI is of great significance, for example, avoiding night shifts in progestation to balance the secretion of melatonin (Wei et al., 2020) and decrease the incidence of AI.

Although the involvement of the signaling pathway associated with melatonin in ameloblastic differentiation and mineralization has not been elucidated, several studies have reported an association between development and the MAP kinase signaling pathway under the influence of melatonin, such as p38 and Prkd1 (Son et al., 2014), Ras/Raf/ERK (Tiong et al., 2020), MEKs (Maria et al., 2018), JNK, NF-κB (Cho et al., 2015), et al. We performed mRNA sequencing on the ALCs to study the complex network evoked by melatonin, and by DEGs analysis, the differential expression of JNK3 and β-arrestin1 was found. JNK3 expression is mainly confined to neurons, pancreas, testis, and the heart (Kant et al., 2019), which is primarily localized in the neurons of Central Nervous System (CNS) and is also the most responsive isoform to stress-stimuli (Chan et al., 2002). So far, the role of JNK3 in the nervous system has been well studied and frequently used as a biomarker for CNS disease diagnosis (Musi et al., 2020). Meanwhile, melatonin is a neuroendocrine hormone, and ameloblasts derived from dental epithelial cells were differentiated from neuroectoderm stem cells. We speculate that there is a certain relationship between JNK3 related MAPK signaling pathway and melatonin. JNK3 expression in ameloblast is not high enough comparing to neural system but caused a radical changes in mineralization. Sparks of neural JNK3 can start a prairie change, because kinases are intracellular signaling enzymes that catalyze the phosphorylation of specific residues in their target substrate proteins (Zeke et al., 2016). The expression of neural JNK3 in enamel forming cells can be interpreted as ameloblastic progenitor cells have migrated from the cranial neural crest along the future alveolar ridge (Simmer et al., 2010), and it preserves the expression characteristics of nerve tissue to some extent, by which neural JNK3 can superiorly responses to the regulation of neurohormone melatonin. JNK3 was originally considered to act primarily in the nucleus through their modulation of transcription factor actions to alter gene expression programs (Tiwari et al., 2011), which can explain the up-regulation of mRNA of EMPs.

**FIGURE 6:**
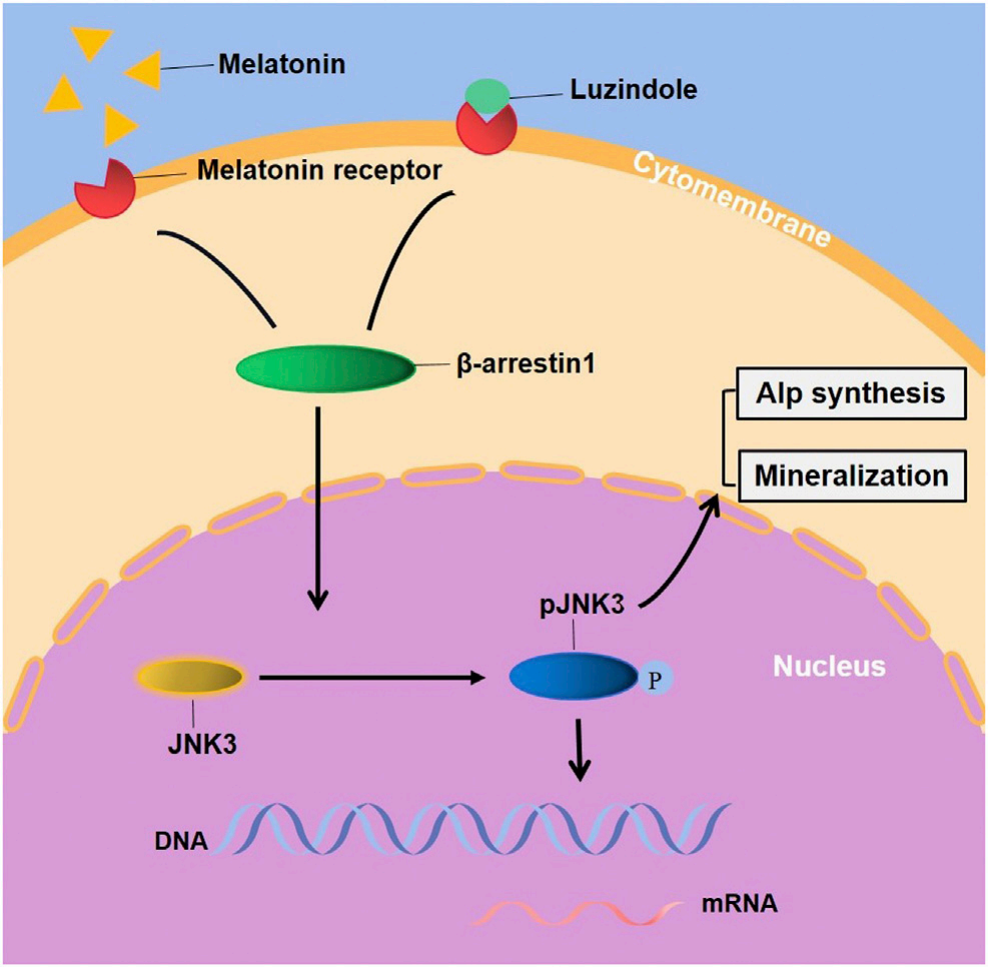
Melatonin stimulated the EMPs transcription, ALP synthesis and mineralization of ALCs through JNK3-Arrb1 pathway. Supplementary Figure. **(A)** The quantified data of Alizarin Red S staining after 1-month melatonin and/or Luzindole odontogenic induction. **(B)** The quantified data of Alizarin Red S staining after 1-month melatonin with/without 25μM/50 μM SR3576 odontogenic induction. **(C)** The gene expression of MTNR1a and MTNR1b in ALCs. **(D)** the protein expression of MTNR1a and MTNR1b in ALCs.

Evidently, G-protein-mediated signaling (MTNRs) is terminated by coupling of β-arrestin1 (Arrb1, arrestin-2), which displaces Gs and induces signaling through the MAPK pathway (Lee et al., 2020). McDonald and colleagues have showed that the MAPK Jun N-terminal kinase 3 (JNK3) is another binding partner of β-arrestin 2. β-arrestins bind to JNK3 and recruit upstream kinases (Ma and Pei, 2007; McDonald et al., 2000). The regulation of kinases is a critical step in signal transduction but there has not been relevant literature reported the linkage between β-arrestin1 and JNK3 signaling pathway. Association with β-arrestins both facilitates activation of the kinase cascade and ensures proper subcellular localization (Lin and DeFea, 2013). A study has reported that prior binding to Ask1 strengthened the interaction of β-arrestin-2 with MKK4 and JNK3 and both MKK4 and Jnk3 phosphorylation are dependent on β-arrestin-2 binding (Li et al., 2019b). Thus, we constructed a stably-overexpressed cell line, results showing that the overexpression of Arrb1 up-regulated the JNK3 both in mRNA and protein levels, enhanced phosphorylation, and facilitated the nuclear localization of JNK3. But the regulatory pattern and correlated factor required further exploration. This suggests that melatonin may regulate ameloblastic mineralization through MTNR-mediated JNK3-Arrb1 signaling pathway (Figure 6). JNK3 and Arrb1 might be used as biomarkers for diagnosis or therapeutic targets for the treatment of enamel hypoplasia, which is possible in the future. There are actually two major action mechanisms of melatonin: MTNR-dependent and MTNR-independent. Melatonin has no corresponding receptor to bind with in MTNR–independent pathway. It can only penetrate cellular membranes and even nuclear membranes to function. The mechanics are quite complex, involving orphan nuclear receptor (Preitner et al., 2002), lipid solubility of melatonin and so on, which need further studies.

In addition to signaling mechanism, the development of enamel is also related to ectoderm-mesenchymal interactions and allelic mutations. In our pending animal experiment and bioinformatic analysis, we will conduct more extensive research.

## Data Availability

The data presented in the study are deposited in the Sequence Read Archive (SRA) of NCBI repository, accession number PRJNA770009.
