# Supplementary material for: Melatonin-Medicated Neural JNK3 Up-Regulation Promotes Ameloblastic Mineralization
Source: Front Cell Dev Biol. 2021 Dec 24;9:749642. doi: 10.3389/fcell.2021.749642 (PMC8740296; doi:10.3389/fcell.2021.749642)
Supplement: Supplementary file 1 [file Table1.DOCX]

**Supplementary1**

Table1

| Name | Forward Sequence(5’-3’) | Reverse Sequence(5’-3’) |
| --- | --- | --- |
| AMBN | GAGACAATGAGACAGTTGGGAAG | GATGGCTGTGATGGGAGAGG |
| AMELX | CCCCAGTCACCTCTGCATC | GCTGCATGGAGAACAGTGG |
| ENAM | GCCCCACCAATGATGCCTAT | TGTGGATTGGTCTGGTTGGG |
| ODAM | ATCAATTTGGATTCGCACCACC | AGTTGGATCTATCCCAGAAGTGA |
| AMTN | CTGTCAACCAGGGAACCACTC | TCCAACTGTGATGCGGTTT |
| TUFT | TGGAGGCTGAGAACTTAGAGATG | GGATGAGAGGCATAGGCTTGG |
| JNK3 | TGGTTCGCCACAAAATCCTC | GGCCGATTCTCCACGTAGTT |
| Arrb1 | AGGCAAGCCCCAATGGAAAG | AGTGTCACGTAGACTCGCCTT |
| β-Actin | GGAGATGGCCACTGCCGCAT | GCAGCTCAGTAACAGTCCGCCTA |

**Supplementary 2**

Table2

| Name | Forward Sequence(5’-3’) | Reverse Sequence(5’-3’) |
| --- | --- | --- |
| Lifr | GGTCTGCTCTGCCTCAC | CTACTGGTTCCGTCCTTGG |
| STAT1 | CTGTCATCCCGCAGAGAGAA | GAGCAGAGCTGAAACGACCT |
| STAT2 | TCCGCTGTTCGCTATCTTGG | TGCGCCATTTGGACTCTTCT |
| Rgl1 | CCTGTTCCTCCATCCATTCC | TGTTGCCATTGTTGTCTTCTAC |
| Slc34a2 | CGTTGCTGCTGCTGTTG | TTCTCCTCCTCTTCCTCTCC |
| Cdkn1a | AGTGTGCCGTTGTCTCTTC | TCAAAGTTCCACCGTTCTCG |
| Dusp4 | CTCTTCAGACTGTCCCAATCAC | CGCTTCTTCATCATCAGGTAGG |
| Rin1 | GTACCTGGGCACCAACAGAA | AGATTTCCGCACCAGGAAGG |
| MAP3K5 | ATTGCTGTGGTGGAGATGAG | GGAGTGAATCGGAATTAGTATCG |
| MAP3K6 | TCCAGAAGAACTCGGATTGTGT | GTAGCCTCCAACAGACGGAC |
