# Supplementary figures and images for: Melatonin-Medicated Neural JNK3 Up-Regulation Promotes Ameloblastic Mineralization
Source: Front Cell Dev Biol. 2021 Dec 24;9:749642. doi: 10.3389/fcell.2021.749642 (PMC8740296; doi:10.3389/fcell.2021.749642)

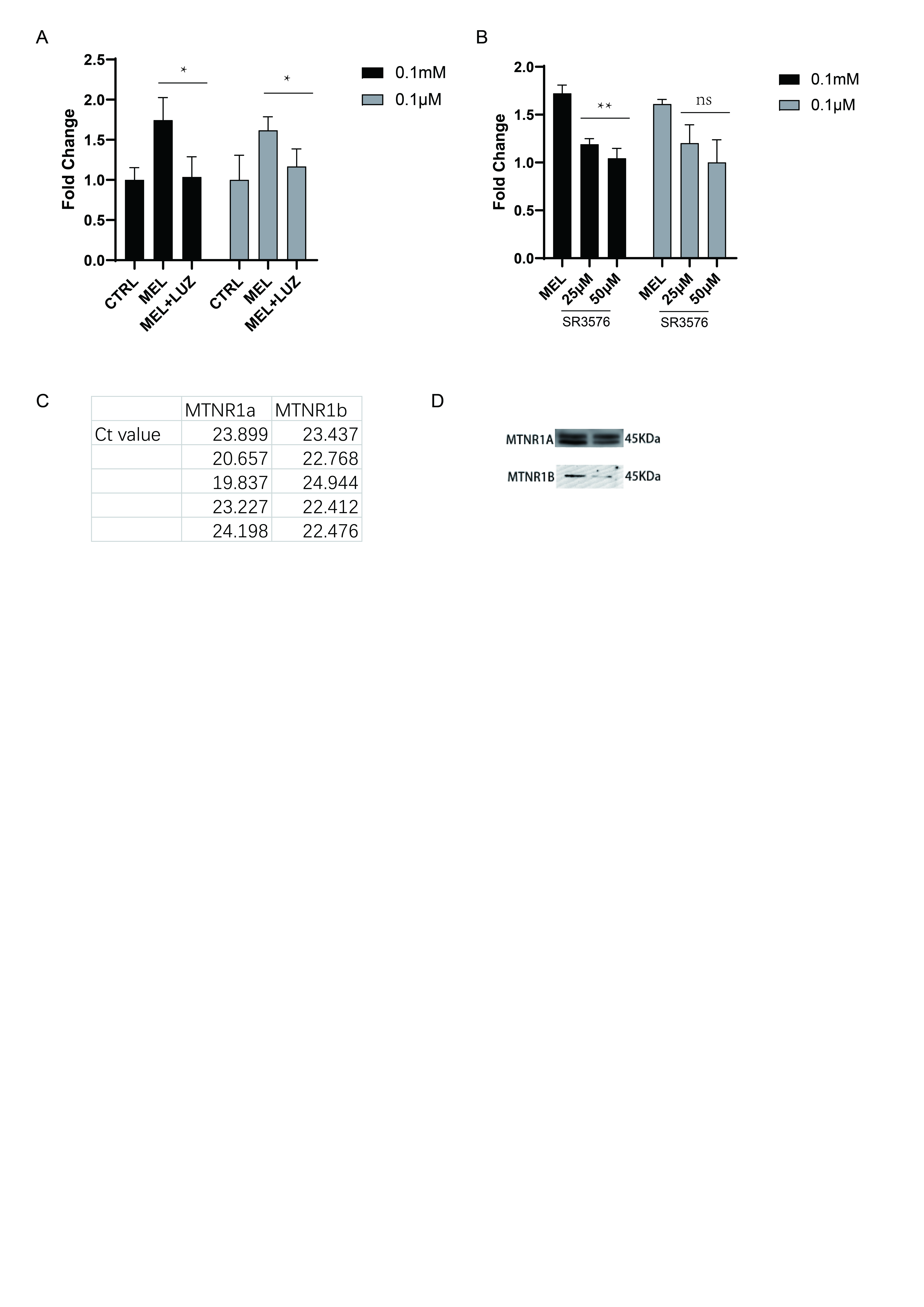

Supplement: Supplementary file 2 [file Image1.JPEG]
